# Skin Dialogues in Atopic Dermatitis

**DOI:** 10.3390/diagnostics12081889

**Published:** 2022-08-04

**Authors:** Elena Porumb-Andrese, Claudia Florida Costea, Andrei Cucu, Gabriela Rusu-Zota, Daciana Elena Braisteanu, Vlad Porumb, Mihaela Monica Scutariu, Alexandra Maria Dorobanțu, Ramona Gabriela Ursu

**Affiliations:** 1 Department Dermatology, Faculty of Medicine, University of Medicine and Pharmacy “Grigore T. Popa”, Universitatii St. No. 16, 700115 Iasi, Romania; 2 Department of Surgery II, Discipline of Ophthalmology, Faculty of Medicine, University of Medicine and Pharmacy “Grigore T. Popa”, Universitatii St. No. 16, 700115 Iasi, Romania; 3 2nd Neurosurgery Clinic, Professor Doctor Nicolae Oblu Emergency Clinical Hospital, 700309 Iasi, Romania; 4 Department of Pharmacology, Clinical Pharmacology and Algesiology, Faculty of Medicine, University of Medicine and Pharmacy “Grigore T. Popa”, Universitatii St. No. 16, 700115 Iasi, Romania; 5 Department of Surgery I, Faculty of Medicine, University of Medicine and Pharmacy “Grigore T. Popa”, Universitatii St. No. 16, 700115 Iasi, Romania; 6 Department of Dental Technology, Faculty of Dental Medicine, University of Medicine and Pharmacy “Grigore T. Popa”, Universitatii St. No. 16, 700115 Iasi, Romania; 7 Dermatology Department, Elias Emergency University Hospital, 011461 Bucharest, Romania; 8 Department of Microbiology, Faculty of Medicine, University of Medicine and Pharmacy “Grigore T. Popa”, Universitatii St. No. 16, 700115 Iasi, Romania

**Keywords:** atopic dermatitis, comorbidities, cardiometabolic risk, neoplasia risk

## Abstract

Atopic dermatitis (AD) is a chronic skin disorder associated with significant quality-of-life impairment and increased risk for allergic and non-allergic comorbidities. The aim of this review is to elucidate the connection between AD and most common comorbidities, as this requires a holistic and multidisciplinary approach. Advances in understanding these associations could lead to the development of highly effective and targeted treatments.

## 1. Introduction

In the past two decades, allergic disorders have become a major global health problem that is currently estimated to afflict between 25% and 40% of the population in the developed world. These diseases, such as atopic dermatitis, asthma, or allergic rhinitis, are associated with significant morbidity and quality-of-life impairment, in addition to presenting a high socioeconomic burden [1].

What does atopy mean? The word is derived from the Greek atopia, meaning “different”. The term “atopy” was originally proposed in 1923 by Coca and Cooke in order to describe asthma and allergic rhinitis, and the concept of atopic dermatitis was introduced 10 years later in 1933 by Wise and Sulzberger [2].

Atopic dermatitis (AD) is one of the most common and complex chronic skin disorders characterized by pruritus and inflammation, with most patients having a personal or family history of “atopic march” [3]. The World Allergy Organization estimates that the disease affects 10–30% of the pediatric population and 2–10% of adults in developed countries, with onset occurring in the first 6 months of life in 45% of affected children and during the first year of life in 60% cases. In terms of prevalence, AD in industrialized countries has tripled in the past 30 years, and although it is more common in young people, AD in most adults continues for many years [4,5].

The most important function of the skin is to provide a first-line defense between the body and the environment. The epidermal barrier serves three primary functions: limiting passive water loss, restricting environmental chemical absorption, and preventing microbial infection [6]. Although the pathophysiology of AD is not completely understood, numerous studies have demonstrated that skin barrier dysfunction is a central feature of AD, and with immune dysregulation as the second contributing factor to the pathobiology. In the acute phase, T helper 2 and T helper 22 responses are increased in the skin, with some implications from T helper 17. The disease continues its progression with an increased role played by T helper 1 and T helper 2 cells. The disease is considered a product of the interplay between environmental factors and genetic susceptibility [7].

**Figure 1:**
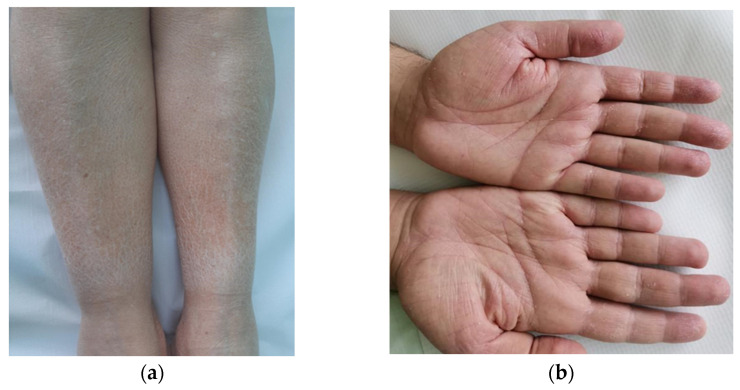
Cutaneous xerosis (**a**); palmar hyperlinearity (**b**).

AD is clinically diagnosed through extensive history and physical examination of the morphology, distribution, and clinical course of lesions. It generally presents three clinical phases: acute (with vesicles, erythema, oedema, and crusts), subacute (scaly and dry skin, papules), and chronic (dyspigmentation, thickening, xerosis and lichenification), although phases may coexist. Pruritus is a hallmark of AD, and excoriations secondary to scratching are often present. Generalized xerosis is a ubiquitous clinical feature that is often associated with palmoplantar hyperlinearity (Figure 1). In 1979, Hanifin and Rajka (H-R) advanced major criteria for this diagnosis; the criteria are comprehensive and considered the “gold standard”. The United Kingdom Working Party criteria are an abridged version of the H-R criteria and tend to work better in children than adults. The American Academy of Dermatology has also developed a streamlined version of the H-R criteria [8].

Given its tremendous burden on physical, mental, and social health, numerous scoring systems have been described to evaluate AD. The scores can be classified into two categories: those aimed at evaluating skin involvement, such as the Eczema Area and Severity Index (EASI) for mild-to-severe AD in both pediatric and adult patients; the Self-Administered Eczema Area and Severity Index (SA-EASI); SCROAD (SCORing Atopic Dermatitis); Patient-Oriented SCOring Atopic Dermatitis (PO-SCORAD); and those aimed at evaluating quality of life, such as the Dermatology Life Quality Index (DLQI), and Patient-Oriented Eczema Measures (POEM). QoL measurement has become an essential aspect of monitoring disease symptoms in AD to assess the safety and efficacy of therapies in clinical trials [9]. Other alternatives are the Hospital Anxiety and Depression Scale (HADS), the Numerical Rating Scale (NRS), and the Visual Analogue Scale (VAS); however, there are no specific tests to define and evaluate AD, and further validation of these methods in AD is needed.

**Figure 2:**
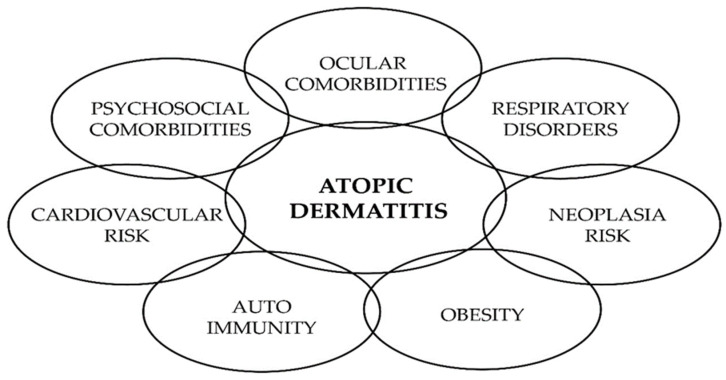
Common comorbidities of atopic dermatitis.

Patients with AD are at high risk of developing allergic and non-allergic comorbidities, and early onset is considered a risk factor in the development of such associations. The relationship between comorbidities and AD is likely bidirectional and multifactorial, reflecting the systemic nature of the disease (Figure 2). Allergic conditions include asthma, allergic rhinitis, atopic keratoconjunctivitis, blepharitis, eosinophilic esophagitis, and food allergy. Non-allergic comorbidities include cutaneous bacterial infections (Staphylococcus aureus colonization and Streptococcus pyogenes), cutaneous viral infections (eczema herpeticum, eczema coxsackium, ezcema vaccinatum, and molluscum contagiosum), or extracutaneous infections, as well as mental health comorbidities (attention-deficit hyperactivity, autistic spectrum disorder, anxiety, and depression), cardiovascular diseases, obesity, autoimmune diseases, or gastrointestinal immune-mediated disorders [7,10]. The mechanisms underlying these associations are mostly unknown, and the absolute magnitude of the risks has not been well defined to date.

## 2. Respiratory Disorders

### 2.1. Allergic Rhinitis

Chronic rhinitis is divided into allergic rhinitis (AR) and non-allergic rhinitis. In a case of AR, patients test positive in a skin prick test or for the presence of IgE in serum. Patients with non-AR test negative for both. Among the atopic disorders, AR is the most prevalent, affecting up to 40% of the population [11]. The disease is defined as chronic inflammation of nasal mucosa driven by type-2 helper T (Th2) cells with symptoms of sneezing, airflow obstruction, nasal itching, and clear nasal discharge [12]. Numerous predisposing risk factors have been described for AR, such as history of allergic diseases, early-life antibiotic use, male sex, maternal smoking, elevated serum IgE levels, and indoor allergen exposure [13].

Epidemiological studies have shown that the presence of AR is an increased risk factor for development of asthma; up to 40% of people with AR have or will have asthma [14].

In terms of therapeutic options, various therapeutic agents are used, such as mast cell stabilizers, chemical mediator receptor antagonists (antihistamines, antileukotrienes, prostaglandin D2, and thromboxane A2 receptor antagonists), Th2 cytokine inhibitors, steroids, and alpha-sympathomimetics [15].

### 2.2. Allergic Bronchial Asthma

AR and bronchial asthma (BA) can be considered as different aspects of one systemic allergic disorder. Generally, the history begins with AD and progresses, in an additive manner, to include asthma and AR, which is defined as a chronic inflammatory disorder of the airways that translates clinically in recurrent episodes of wheezing, chest tightness, or coughing [16].

A positive correlation has been demonstrated between the severity of AD and the risk of developing bronchial asthma (BA). In a large study of 3225 AR patients, Valero et al. examined this relationship and concluded that respiratory allergic disease is a systemic condition and that some characteristics of AR may affect the development of asthma [17].

On the other hand, in middle-income countries, studies have suggested that atopic sensitization it is not strongly correlated with sequential development of AD, BA, and AR. A possible explanation for this relationship is that environmental exposures in late childhood may dissociate atopic conditions from each other [18,19].

## 3. Psychological Distress in Atopic Dermatitis

Recent studies have also shown that an important pathogenic role is played in illness development by emotional stress and various personality traits. Both acute and chronic stress exacerbate disease symptoms by altered innervation of the skin and elevated tonus of the sympathetic nervous system [20,21].

Associations have also been recognized between allergic diseases and psychiatric disorders, such as depression, anxiety, and, perhaps the best-studied mental health comorbidity associated with ADHD, attention deficit/hyperactivity disorder [22]. The association between AD and ADHD is unsurprising, given the Diagnostic and Statistical Manual of Mental Disorders, 5th edition, diagnostic criteria for ADHD, with characteristic features that overlap with those of an itchy child. The chronic use of antihistamines, an often-used therapy in AD, was significantly also associated with increased ADHD symptoms [23]. In a German study, it was shown that the risk of depression for these patients is comparable with the risk of depression in patients suffering from cancers [24]. In a Swedish study with 34,313 participants, adults with AD were found to have an increased risk of severe depression, anxiety, and reduced quality of life [25]. The specificity of the relationship remains unclear, but a recent meta-analysis highlights a two-way direction between mental health and atopic disorder [26].

A recent study from Korea that included 72,435 adolescent middle and high school students with AD revealed that AD patients are prone to depressive feelings, as well as ideation and planning attempts at suicidal behaviors [27].

## 4. Ocular Comorbidities

Ocular complications of atopic dermatitis include blepharitis, conjunctivitis, modifications of the lacrimal canaliculi, cataract, uveitis, keratoconus, and retinal detachments [28,29].

Keratoconus was described by Hilgatner in 1937 as atopic dermatitis complication; patients diagnosed with keratoconus have symptoms of eye rubbing [30]. Bawazeer et al. mentioned that eye rubbing occurs in the cases of progressive keratoconus and atopy may contribute to keratoconus probably by eye rubbing [31]. Allergic disorders (atopic keratoconjunctivitis, asthma, and atopic dermatitis) are also associated with corneal hydrops in keratoconus [32].

Bacteria that colonize eyelid skin and conjunctival fornixes are more numerous in patients with atopic dermatitis than in healthy individuals. The most frequently identified pathogen in patients with atopic dermatitis is Staphylococcus aureus (67%) [33].

Other ocular complications during atopic dermatitis include blepharitis, retinal detachment, subcapsular cataract, keratoconjunctivitis, uveitis, and ocular herpes simplex. Retinal detachment is a serious complication of atopic dermatitis and is often caused by eye rubbing [34,35,36,37,38].

Cataract in atopic dermatitis is usually bilateral, with lens opacities in the anterior and posterior subcapsular region [39]. Sasabe et al. proved the association between atopic dermatitis in patients with cataract and high levels of IgE [40].

Patients with severe atopic dermatitis were associated with advanced glaucoma induced by glucocorticosteroids, and eye rubbing in these patients may be a cause of ocular trauma, possibly causing globular rupture [39,41].

In the case of children, in a study of 59 children with atopic dermatitis, Carmi et al. observed several associated eye diseases, including chronic blepharitis (1 case), papillofollicular conjunctivitis (11 cases), purulent bacterial conjunctivitis (1 case), nuclear cataract (1 case), and amblyopia (1 case) [35].

### 4.1. Atopic Keratoconjunctivitis (AKC)

Atopic keratoconjunctivitis (AKC) is another comorbidity of AD characterized by redness, ocular pruritus, burning discomfort, pain in the eye, or photophobia. AKC is a noninfectious, inflammatory condition that affects the cornea and conjunctiva and occurs in an estimated 25% to 42% of patients with AD [31]. AKC can progress to recurrent corneal erosions, corneal ulcers, keratoconus, cataracts, blepharitis, tear dysfunctions, or infectious keratitis. It is also a potentially blinding condition if not treated early [41,42,43,44,45].

Atopic keratoconjunctivitis was the most common ocular manifestation identified in children diagnosed with atopic dermatitis [45].

**Figure 3:**
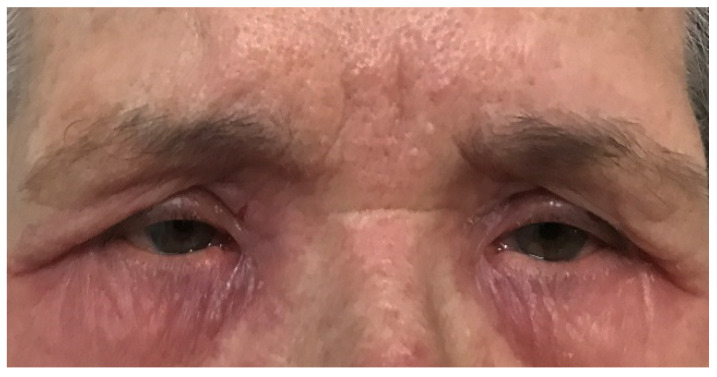
Periocular atopic dermatitis (PD).

The diagnosis of atopic dermatitis in adult and pediatric patients requires ophthalmological check-ups in order to identify possible associated eye diseases (Figure 3).

Treatment of atopic dermatitis is essential in the prevention of eye rubbing, which can cause severe eye complications, such as retinal detachment or worsening of the keratoconus, causing permanent impairment of visual acuity. A recent study showed that patients with PD treated with oral antihistamines and topical steroids were approximately four times more likely to experience cure than those treated with topical steroids alone [46]. Topical calcineurin inhibitors are another highly effective approach for treatment of conjunctival involvement [47]. This could underline the importance of allergy testing for this category of patients.

### 4.2. Blepharitis

Blepharitis is an inflammatory condition of the eyelids that is estimated to affect more than 6% of patients with AD. Clinically, patients may experience pruritus and local irritation, tearing, burning and foreign sensation, photophobia, or crusting of the eyelids [48].

## 5. Immune Dialogue between the Gastrointestinal Tract and Skin

The role of gut microbiota in the development and severity of symptoms of AD remains unclear. However, it has been proposed that probiotics may lead to an induction of regulatory of T cells, along with suppression of regulatory cytokines Il-10 and TGF-β [49]. Systemic antibiotic treatment has been reported to increase the risk of AD, possibly due to changes in intestinal microbiota [50].

Other studies revealed a significant reduction in the microbial diversity of the oral cavity in AD patients, as well as an apparent alteration in genera [51].

More than 90% of all microorganisms in humans are found in the large intestine. Previous studies have revealed that changes in the gut microbiome in early life are closely correlated with age of onset and severity of AD; abundant evidence suggests that host-microbiome interactions can determine health status. The development of skin changes may be promoted not only by gut microbiome diversity but also by specific interactions between the gut microbiome, the immune system, and the host [52]. In patients with AD, *Clostridium difficile*, *Escherichia coli*, and *Staphylococcus aureus* levels in the gut microbiome are higher than in healthy controls [53]. Transepidermal water loss decreased in human subjects after oral supplementation of *Lactobacillus brevis* SBC8803 [54]. Transepidermal water loss is an indicator of skin barrier function. In animal models of AD, infection with *Helicobacter pylori* exerts a protective effect on the severity of skin lesions, such as erythema, swelling, and hyperemia [55]. The potential for probiotics to treat inflammatory skin diseases has been a focal point of research. For example, a number of randomized, controlled trials have reported a reduction in disease severity following administration of oral probiotics to children with AD [56,57]. Conversely, other studies have noted no differences between oral probiotics and placebo in preventing or improving AD symptoms [58].

Another study using *Lactobacillus acidophilus* L-92 in children between 10 months and 3 years old who had undergone conventional treatment and with concomitant food allergies (FA) showed a decrease in allergic sensitization, with decreased SCORAD index and decreased total IgE levels after only 24 weeks. These results suggest that L-92 works as an adjunctive treatment in patients with AD and FA [59].

Irritable bowel syndrome, a functional gastrointestinal disorder characterized by chronic or recurrent abdominal pain, cramping, gas, bloating, and diarrhea/constipation, was found to be more common in AD patients that in healthy controls in a recent study conducted by Kaya İslamoğlu et al. The relationship could be explained by inflammation, an important factor in the pathogenesis of both diseases, with elevated serum inflammatory cytokines and decreased serum anti-inflammatory cytokines for irritable bowel syndrome. In adults, risk of celiac disease was also increased but not in children [60].

## 6. Cardiometabolic Risk

Until now, AD has been linked to comorbidities such as allergies, asthma, and psychiatric disorders; however, recent studies have suggested an association between AD and cardiovascular disease in both adults and pediatric patients. Cardiovascular comorbidities for AD include hypertension, myocardial infarction, stroke, angina, coronary heart disease, heart failure, peripheral artery disease, thrombosis, and atrial fibrillation. The link between atopy and cardiovascular risk is based on the theory of a shared inflammatory pathway [61].

A meta-analysis of 30 studies showed higher odds of overweight or/and obesity in children and adults with AD in studies from North America and Asia [62]. A Taiwanese cohort study showed a significantly increased risk of risk of ischemic stroke but not of myocardial infarction (MI) in patients with AD [63]. In Europe, a recent large German study revealed a modestly increased risk of hypertension, angina, and peripheral artery disease but not of MI and stroke [64].

Compared to other chronic inflammatory skin conditions, such as psoriasis, the risk for cardiovascular diseases in AD is much lower, although it remains controversial.

A possible link between AD and type 2 diabetes is likely multifactorial and possibly explained by genetic predisposition; AD-associated therapies, including corticosteroids; lifestyle; or systemic low-grade inflammation [65,66]. In contrast, children diagnosed with type 1 diabetes mellitus seem to be less likely to develop AD compared with controls; however, evidence of this association is limited, and large control studies are needed to elucidate the exact link between AD and diabetes [67]. Nevertheless, it is difficult to determine whether the potential association of AD with these types of comorbidities is due to pathophysiological effects, adverse events associated with medications used to treat AD, or a combination of the two factors.

### Obesity

A recently meta-analysis of epidemiological studies showed that obesity is associated with an increased prevalence of AD in both children and adults. Such an association can be interpreted in three ways: obesity causes AD by providing chronic inflammatory status; obesity can influence sex hormones by increasing 17-β estradiol plasma concentrations, and estrogens have proinflammatory properties; or poor diet habits or sedentary lifestyle could lead to both illness [61].

The association between obesity and atopy may be linked to genetics or shared risk factors, such as gut microbiome. However, the reported results are conflicting. For example, a large study with more than 15,000 children showed that excessive gestational weight gain was associated with a 10% increase in risk of AD [68], whereas another study revealed that obese children younger than 2 years were 15 times more likely to have the disease [69].

Obesity is known to modify cutaneous and systemic inflammation via increased cytokines secretion (tumor necrosis factor alpha and interleukin 6) [70].

These molecules may predispose patients to hypersensitivity reactions. Another explanation could be the predisposition to a more sedentary lifestyle, which may increase the risk of obesity [71].

A cross-sectional analysis of a large population-based cohort (441,746 adults with AD) revealed a small association between AD and overweight and obese patients but no association with severe forms of obesity [72]. An interesting aspect is that these studies used body mass index to determine obesity rather than measurements of abdominal obesity/waist circumference, possibly resulting in a stronger association between AD and obesity. However, the authors drew the common conclusion that maintaining ideal body mass index during early childhood important aspect for patients with atopic march. A cross-sectional study of 116,816 patients from Israel revealed that metabolic syndrome to was less prevalent in people with AD, whereas another study from Korea revealed this association only in female subjects. In general, females are more prone to severe allergies because estrogen is generally known to enhance the activity of eosinophil and inhibit the production of cortisol [73].

## 7. Cancer and Atopic Dermatitis

The association between cancer and atopic dermatitis remains controversial. A recent meta-analysis showed that there was a small but significantly increased risk of lymphoma in the AD cohort and that AD severity is a significant risk factor [74]. On the other hand, a meta-analysis found no association with acute myelogenous leukemia in two studies and an inverse association between AD and acute lymphoblastic leukemia in six studies [75]. In a prospective adult Dutch cohort study, Hajdarbegovic et al. found an inverse association between AD and actinic keratosis but no association with non-melanoma skin cancer (NMSC) [76]. In a recent systematic review, AD was found to be associated with a significantly elevated risk of NMSC but with a non-significantly decreased risk of melanoma. However, Karim et al. showed that allergic diseases appeared to reduce the risk of developing skin cancers [77,78]. No association was found between AD and brain, pancreatic, or bladder cancer [79].

The relationship between allergies and cancer is based on four hypotheses: chronic inflammation, inappropriate T-helper 2, immune-skewing immunosurveillance, and prophylaxis [80].

## 8. Cutaneous Infections

AD is associated with cutaneous bacterial infections and more severe forms of cutaneous viral infections. A recent large study concluded that pediatric and adult AD patients were associated with significantly higher odds of erysipelas, impetigo, carbuncle/furuncle, cellulitis, methicillin-resistant and methicillin-sensitive Staphylococcus aureus infections, herpes simplex, molluscum contagiosum, human papillomavirus, zoster virus, dermatophytosis, and candidiasis of the skin/nails [81].

### 8.1. Bacterial Infections

Approximately 80–90% of patents with AD are colonized with *S aureus*, which produces enterotoxins that are known to break down the skin barrier and enhance type 2 inflammation [82]. Clinically, features that suggest secondary bacterial skin infection are extensive disease, and oozing, crusting, or asymmetrical lesions [83]. Doctors should also be aware of the existence of a rare syndrome that associates dermatitis with recurrent skin infections. Job syndrome is a rare, primary immunodeficiency characterized by the clinical presentation of AD, staphylococcal infections, and recurrent pulmonary infections. The disease is distinguished by elevated IgE levels with an early onset in primary childhood and is classified into two types: type I (autosomal dominant hyper IgE syndrome), with abnormalities in multiple systems, the including skeletal, dental, and immune systems; and type II (autosomal recessive hyper IgE syndrome), with no musculoskeletal alterations [84].

Recent studies have shown that local treatment with topical corticosteroids, emollients, and bleach baths resulted in decreased *S. aureus* and increased microbial diversity, including *Corynebacterium*, *Propionibacterium*, or *Streptoccocus*. Fundamental knowledge about RNA, in association with the considerable technological advances in recent years provided by cutting-edge technologies in molecular biology, has led to biotechnological and medical applications of RNA, including in AD. We identified some interesting studies (see Table 1) which using mRNA analysis for different bacterial species to optimize AD therapy [85,86,87,88,89].

### 8.2. Viral Infections

Approximately 20% of patients with AD develop eczema herpeticum, an acute, life-threatening viral infection caused by herpes simplex virus [90]. Recurrence was associated with an earlier onset of AD but not with total IgE serum levels. This form of Herpes *simplex* infection is positively associated with onset of AD at an early age, more severe disease, greater T-helper 2 polarity, and other skin infections [91]. Molluscum contagiosum is another viral infection frequently associated with higher recurrence of AD flares is some pediatric patients, who are more likely to have a family history of atopy [92].

### 8.3. Fungal Infection

*Malassezia* spp. is common in AD patients, with sensitization rates particularly higher in cases with head and neck types of AD. Some recent studies investigated a possible correlation between IgE-mediated sensitization to *Malassesia* spp. and disease severity. This correlation seems to be more frequent in adults than in children [93].

Filaggrin mutations could increase the risk of *human papilloma virus* in the cervical mucosa. Immunosuppressive drugs used to treat AD were investigated with respect to the associated risk of this type of infection. It seems that AD patients treated with oral immunosuppressive therapy have no additional risk of developing cervical neoplasia [94].

Children with AD have a higher prevalence of extracutaneous infections, such as streptococcal pharyngitis, recurrent otitis, urinary tract infections, and infective endocarditis [95]. Most recently, the impact of COVID-19 on patients with AD was established by specific questionnaires that highlighted the heavy burden placed on patients during such periods. Predictive factors were identified, such as sex (females tend to be more vulnerable) and age (individuals under 40 years old). Reports also suggest that lower education level, unemployment, and poor economic status were significant risk factors for developing depressive symptoms during the pandemic period in the general population [96].

In terms of dermatologic conditions, AD has been associated with alopecia areata, vitiligo, dermato/polymyositis, cellulitis, actinic keratoses, systemic lupus erythematosus, connective tissue disease, Sjogren syndrome, and seborrheic dermatitis [97].

In a recent study that included 70,584 individuals with AD and 270,783 controls, eating disorders were positively associated with AD, with the highest association with bulimia nervosa, followed by binge eating disorders [98].

Comorbidities were shown to differ according to geographical region, for example, cardiovascular comorbidities were less registered in Europe (large German, Danish, and Canadian investigations) that in the USA. This could be explained by different therapeutic options available in different countries, divergent diagnostic criteria, age of patients, or lifestyle factors (e.g., smoking and diet) [99]. Other studies have shown that children had a higher prevalence of infections, whereas adults had a higher prevalence of mental disorders [100]. Large variations in the prevalence of comorbidities in countries with ethnically homogenous populations could be explained by the fact that, alongside genetic predisposition, environmental factors play an important role in the pathogenesis of the disease.

## 9. Conclusions

Recent studies have redefined the concept of AD, which is no longer considered a disease that affects only the skin but a systemic illness; therefore, the concept of classic atopic may be further expanded. The causative mechanisms underlying AD and its associated comorbidities are largely unknown, but many of these conditions are directly connection with patient age, chronicity, or inadequate disease control. There is a growing list of potential comorbidities that suggests that patients should be treated with a multidisciplinary approach. Understanding these comorbidity patterns can help us to ensure better care of patients with AD. AD could be considered a systemic inflammatory disease with multiorgan involvement beyond the skin. Recent advancements in the understanding of the molecular pathogenesis are closely related to the development of therapeutic agents specifically targeting type 2 inflammatory pathways. Important is to mention the role of biologic drug dupilumab; its approval for the treatment of AD and of different type 2 immune mediated conditions led to the concept of treating skin manifestations together with comorbidities in AD. The introduction of dupilumab and of other recently approved targeted drugs or those in advanced stages of development has been made possible by advances in understanding of molecular mechanisms.

## Figures and Tables

**Table 1:** Associations between AD and bacterial mRNA analysis.

Authors, Year, Country	Sample Type/Objective of the Study	mRNA Analysis Assay	Results	Novelty
Nakatsuji, T. et al., 2021, CA, USA	The safety and mechanisms of action of *Staphylococcus hominis* A9 (ShA9), a bacterium isolated from healthy human skin, as a topical therapy for AD.	Expression of mRNA for psmα	Improvement in local eczema severity was suggested by post hoc analysis of participants with S. aureus directly killed by ShA9.	The safety and potential benefits of bacteriotherapy for AD.
Jeong, D.Y. et al., 2020, Sunchang, South Korea	The efficacy of *Pediococcus acidilactici* SRCM102024 (PA) for treatment of AD in HaCaT cells and NC/Nga mice.	mRNA expression of PAR-2, TNF-α, IL-4, and IL-13	Oral treatment of PA relieved AD symptoms in a dose-dependent manner, preventing overactivation of the immune response.	Oral PA intake may be a safe and effective alternative therapy for AD.
Moran, M.C. et al., 2019, New York, USA	Virulence factors can be directly detected at the protein level from human samples.	mRNA analysis	Staphylococcal virulence factors can be quantified at the protein level directly from skin swabs of atopic dermatitis patients.	Secreted virulence factors are present on the skin of atopic patients, as well as the physiological impact of *S. aureus* in inflammatory diseases, such as atopic dermatitis.

## Data Availability

Not applicable.
